# Obesity and the Development of Lung Fibrosis

**DOI:** 10.3389/fphar.2021.812166

**Published:** 2022-01-10

**Authors:** Xia Guo, Christudas Sunil, Guoqing Qian

**Affiliations:** Department of Cellular and Molecular Biology, The University of Texas Health Science Center at Tyler, The University of Texas at Tyler, Tyler, TX, United States

**Keywords:** obesity, lung, fibrosis, inflammation, high-fat diet

## Abstract

Obesity is an epidemic worldwide and the obese people suffer from a range of respiratory complications including fibrotic changes in the lung. The influence of obesity on the lung is multi-factorial, which is related to both mechanical injury and various inflammatory mediators produced by excessive adipose tissues, and infiltrated immune cells. Adiposity causes increased production of inflammatory mediators, for example, cytokines, chemokines, and adipokines, both locally and in the systemic circulation, thereby rendering susceptibility to respiratory diseases, and altered responses. Lung fibrosis is closely related to chronic inflammation in the lung. Current data suggest a link between lung fibrosis and diet-induced obesity, although the mechanism remains incomplete understood. This review summarizes findings on the association of lung fibrosis with obesity, highlights the role of several critical inflammatory mediators (e.g., TNF-α, TGF-β, and MCP-1) in obesity related lung fibrosis and the implication of obesity in the outcomes of idiopathic pulmonary fibrosis patients.

## 1 Introduction

In the past three decades, the prevalence of obesity has been increasing worldwide. Those obese people suffer from a range of respiratory complications, including asthma, airway hyperresponsiveness, chronic obstructive pulmonary disease, and lung fibrosis (Dixon and Peters, 2018; Hales et al., 2018). The influence of obesity on the lung is multi-factorial that involves both mechanical injury and the roles of various inflammatory mediators produced by excessive adipose tissues and infiltrated immune cells. Adiposity causes increased production of inflammatory mediators, for example, cytokines, chemokines, and adipokines, both locally and in the systemic circulation, and thereby rendering susceptibility to respiratory diseases and altered responses. The effects of obesity on lung function (Salome et al., 2010; Dixon and Peters, 2018), asthma (Peters et al., 2018), and chronic obstructive pulmonary disease (COPD) (Hanson et al., 2014) have been reviewed previously and will not discussed here. In this review, we provide updates on the link between obesity and lung fibrosis with a focus on the critical role of several inflammatory mediators implicated in the development of lung fibrosis associated with obesity.

## 2 Association of Obesity With Lung Fibrosis

Obesity contributes to the fibrosis of different organs including the lung (Pessin and Kwon, 2012), the liver (Chiang et al., 2011), the heart (Cavalera et al., 2014), the kidney (Deji et al., 2009; Laurentius et al., 2019; Liu et al., 2019), and the adipose tissue (Buechler et al., 2015; Marcelin et al., 2019). Pulmonary fibrosis is the progressive scarring of the lung, leading to lung structure remodeling, and respiratory functional impairment. High intake of saturated fatty acids and meat increases the risk of idiopathic pulmonary fibrosis (IPF) (Miyake et al., 2006), a most common form of interstitial lung disease. Observational studies suggest a significant link between obesity and IPF (Miyake et al., 2006; Lee et al., 2014; Pessin and Kwon, 2012). One study shows that IPF patients are approximately twice more frequent to have obesity compared to normal control subjects (Lee et al., 2014). In addition, several risk factors for IPF such as gastro-esophageal reflux, obstructive sleep apnea, and diabetes mellitus are also closely associated with obesity (Anand and Katz, 2010; Zaman and Lee, 2018). Chronic inflammation is considered important in the pathogenesis of lung fibrosis through regulating production of cytokines/chemokines (e.g., tumor necrosis factor-alpha (TNF-α), Interleukins (ILs), and monocyte chemoattractant protein-1 (MCP-1), etc.) and growth factors (e.g., transforming growth factor beta (TGF-β), connective tissue growth factor, and platelet-derived growth factor, etc.) from macrophages and modulating matrix (e.g., via MMP-2), vasculature, growth-factor receptor, and oxidative stress status (Bringardner et al., 2008; Sgalla et al., 2018). Increasing *in vivo* evidence has linked diet-induced obesity (DIO) with lung fibrosis, as determined by collagen deposition or hydroxyproline content (Naura et al., 2009; Ge et al., 2013; Yu et al., 2013, Song et al., 2015; Baack et al., 2016; Chu et al., 2019; Park et al., 2019; Vedova et al., 2019; Han et al., 2021; Hegab et al., 2021) (Table 1). Different inflammatory mediators as well as immune cells are involved in high-fat diet (HFD)-induced lung fibrosis. Recently, vitamin D deficiency has also been suggested as a link between obesity and pulmonary fibrosis. HFD decreased serum 25-hydroxyl vitamin D level and induced lung fibrosis as well as TGF-β1 and phosphorylated Smad2/3 in the lungs of mice. Vitamin D supplementation attenuated these changes caused by HFD in C57BL/6 mice. *In vitro* treatment of bronchial epithelial cells BEAS-2B with vitamin D also suppressed TGF-β1 protein expression (Han et al., 2021). The induction of lung fibrosis by diet also seems to be time dependent. For example, feeding mice with a HFD rich in palmitic acid for 2 weeks did not induce obvious pulmonary inflammation or fibrosis although it promoted bleomycin induced pulmonary fibrosis (Chu et al., 2019). Our recent work shows that a high-fat and high-fructose (HFHF) diet promotes inflammatory infiltration in the lungs of C56BL/6 mice after 10- and 20-weeks’ treatment. Conversely, collagen deposition in the lung tissues as determined by Masson’s staining and Western blotting analysis was found to be prominent only after 20 weeks but not 10 weeks (Qian et al., 2021).

**TABLE 1:** Summary of studies linking obesity and pulmonary fibrosis.

Author (year)	Study model	Main findings associated with HFD
Ge et al. (2013)	SD/HFD feeding of C57BL/6 mice for 12 weeks (60% kcal from fat), followed by challenge with cockroach allergen.	Elevated lung TGF-β, PAI-1 expression, lung collagen expression, and decreased lung function. Infiltrated immune cells, epithelial, and endothelial cells were found as a major source of TGF-β expression.
Park et al. (2019)	SD/HFD feeding of C57BL/6 mice for 12 weeks (60% kcal from fat).	Increased insulin resistance, AHR, peribranchial and perivascular fibrosis, and macrophages in the BAL; insulin stimulates TGF-β1 expression in bronchial epithelial cells *in vivo* and *in vitro*; Anti-TGF-β1 antibody attenuated HFD-induced lung fibrosis.
Hegab et al. (2021)	SD/HFD feeding (60% kcal from fat) for 4 weeks, followed by bleomycin challenge and examine after 3, 6, and 9 weeks.	No significant differences in inflammation and fibrosis severity between SD and HFD-fed flies after 3 weeks; HFD-induced a delay in alveolar repair and fibrosis resolution at 6 weeks following bleomycin treatment.
Naura et al. (2009)	SD/HFD feeding for 12 weeks (42% kcal from fat) in ApoE−/− mice; direct intratracheal TNF-α (100 ng/mouse) administration in ApoE−/− mice for 6 and 24 h.	Elevated TNF-α, IFN-γ, and MIP-1α and increased tissue distribution of TGF-β, recruitment of monocytes and macrophages, subepithelial, and peri-vascular collagen deposition and thickening in lungs of ApoE−/− mice. TNF-α induced proinflammatory cytokines similar as that of HFD and induced MCP-1, TGF–β1, IL-1β, and collagen type 1 expression.
Yu et al. (2013)	HFD (Lieber-DeCarli liquid diet) feeding of male Sprague-Dawley (SD) rats for 8 weeks	Increased TGF-β expression in lung tissues and deposition of collagen fibers at alveolar septa in HFD group.
Han et al. (2021)	SD/HFD feeding of C57BL/6 mice for 12 weeks (60% kcal from fat)	Increased BAL cell numbers; elevated lung TGF-β; increased collagen deposition, hydroxyproline content, and fibrosis; and decreased serum 25-hydroxyl vitamin D in mice received HFD.
Vedova et al. (2019)	SD/HFD (22% chicken fat) and with or without 10% fructose for 16 weeks.	HFD plus fructose additively enhanced pulmonary inflammation, oxidative stress, and pro-fibrotic changes.
Chu et al. (2019)	SD/HFD feeding of C57BL/6 mice (42% kcal from fat) for 2 weeks followed by bleomycin challenge (1.2 mg/kg) via oral aspiration. Lung tissues examined at 3, 7, and 21 days after bleomycin.	HFD for 5 weeks did not induce hydroxyproline in the lung, however, it significantly enhanced bleomycin-increased hydroxyproline content, mRNA levels of collagen 1 and fibronectin at 21 days. HFD induced apoptosis and a prolonged ER stress after bleomycin.
Baack et al. (2016)	Female SD rats fed HFD (40% kcal from fat) 4 weeks before mating, offspring examined at 3 weeks of age.	No difference in lung fibrosis in offspring at 3 weeks of age; increased perinatal mortality and decreased pulmonary vessels in maternal HFD-exposed offspring.
Song et al. (2015)	Female SD rats were fed a SD/HFD for 8 weeks, then bred with normal male rats, maintained on SD/HFD during pregnancy and lactation.	Maternal HFD exposed offspring showed significantly increased pulmonary inflammatory infiltration, collagen deposition, and increased TGF-β and α-smooth muscle actin expression in the lung.

Note: AHR, airway hyperreactivity; BAL, bronchoalveolar lavage; SD, standard diet; HFD, high-fat diet.

The impact of maternal exposure to HFD on the development of lung in offspring is of emerging interest. Maternal exposure to high-fat diet has been shown to impair lung development in the rat offspring, however, no significant changes in fibrosis was observed in offspring at 3 weeks after birth (Baack et al., 2016). In contrast, another study by Song et al. reported opposite results (Song et al., 2015). Maternal Sprague-Dawley rats were given either high-fat diet or standard diet after weaning and throughout pregnancy and lactation. The offspring were maintained on regular diet and evaluated for the lung histology after 3 months. Offspring with maternal HFD feeding showed increased body weight at birth and at 3 months, enhanced inflammatory infiltration and collagen deposition as well as TGF-β1 expression in the lungs. The discrepancy might relate to the different timing of examination after birth. The effect of maternal obesity on lung fibrosis of offspring remains less understood and further research is needed.

## 3 Role of Inflammatory Mediators in Diet Induced Lung Fibrosis

### 3.1 Tumor Necrosis Factor-α

Mounting evidence show the elevated TNF-α in the adipose tissue and the lung in high-fat diet induced obesity. TNF-α plays a critical role in mediating insulin resistance associated with high-fat diet and targeting TNF-α increases insulin sensitivity (Hotamisligil et al., 1993; Tzanavari et al., 2010). The signaling of TNF-α through its receptors seems to be essential in pulmonary fibrosis development. Knockout of TNF-α receptor provides protection of mice against pulmonary fibrosis caused by bleomycin, asbestos, and silica (Liu et al., 1998; Ortiz et al., 1998; Ortiz et al., 1999). In addition, blocking TNF-α with quenching antibody attenuates pulmonary fibrosis induced by bleomycin and silica in mice (Piguet et al., 1989; Piguet et al., 1990). Soluble TNF-α was thought important for transition from inflammation to fibrosis in the lung that involves recruitment of lymphocytes (Oikonomou et al., 2006). In accord, a soluble receptor for TNF-α was shown to alleviate bleomycin-induced pulmonary fibrosis (Piguet and Vesin, 1994). Therefore, endogenous TNF-α and its receptors seem to be required for the development of lung fibrosis.

A previous study showed that overexpression of TNF-α induces mild pulmonary fibrosis in rats (Sime et al., 1998). Similarly, direct intratracheal administration of TNF-α into wild-type mice also results in the expression of ICAM-1, VCAM-1, IL-1β, MCP-1, as well as TGF-β1, and collagen type I (Naura et al., 2009), a pattern of changes in the lung resembling that found in ApoE−/− mice fed a high-fat diet, suggesting a potential role of TNF-α in lung injury, and fibrosis associated with obesity. On the other hand, conflicting evidence suggest an anti-fibrotic role of TNF-α in different contexts. For instance, the transgenic TNF-α mice that overexpress murine TNF-α under the control of the human surfactant protein C promoter (Miyazaki et al., 1995) are more tolerant to bleomycin or active TGF-β1 induced pulmonary fibrosis (Fujita et al., 2003). Administration of recombinant human TNF-α also attenuates bleomycin-induced pulmonary fibrosis in mice (Fujita et al., 2003). In addition, intratracheal delivery of TNF-α facilitates the resolution of established pulmonary fibrosis induced by bleomycin (Redente et al., 2014). The author proposed that locally increased TNF-α might cause apoptosis of profibrotic macrophages, important in pathogenesis of pulmonary fibrosis through secretion of TGF-β1, IGF-1, PDGF, and arginase I. These microphages could also contribute to the pool of myofibroblasts via trans-differentiation (Gibbons et al., 2011). The controversial role of TNF-α in lung fibrosis has been discussed previously (Distler et al., 2008). Some clues might help understand this discrepancy, e.g., the stage-specific changes in inflammation profile (early-stage inflammation versus late-stage resolution and fibrosis), the requirement of inflammation and importantly, TNF-α, in the development of fibrosis, and the direct induction of TGF-β1 by TNF-α. The decision of complete resolution of inflammation or progression towards fibrosis remains unclear and further research is needed. The role of TNF-α in pulmonary fibrosis in the context of diet-induced obesity remains an open question and further research using genetic modified mice or approaches to manipulate the expression of TNF-α in the lung is deserved.

### 3.2 Transforming Growth Factor-β

#### 3.2.1 Induction of Transforming Growth Factor-β by High-Fat Diet

In obesity, increased levels of TGF-β1 in the adipose tissue and/or in the lung have been observed in obese humans, mice, rats, and drosophila (Samad et al., 1997; Yadav et al., 2011, Jung et al., 2013; Sousa-Pinto et al., 2016; Lee, 2018; Park et al., 2019) (Table 1). High-fat diet has been shown to induce TGF-β1 expression in the bronchial epithelium (Park et al., 2019). This finding was thought to be related with insulin resistance, which is closely associated with high-fat diet in mammalian models. The authors demonstrated the induction of TGF-β1 by insulin in BEAS-2b cells and *in vivo* through intranasal administration of insulin to mice fed standard diet (Park et al., 2019). These findings implicate insulin in high-fat diet induced TGF-β1 expression in the lung. Activated macrophages in adipose tissue also contribute to the elevated TGF-β1 in obesity (Chow et al., 2005; Spencer et al., 2010). An alternative programmed macrophage, M2 macrophage, were found increased in fibrotic areas in adipose tissues from insulin-resistant subjects. Those macrophages express higher TGF-β1 and can be further promoted by co-culture with adipocytes (Spencer et al., 2010).

In addition, a role of TNF-α in direct induction of TGF-β1 has been suggested based on the findings that TNF-α induces mRNA levels of TGF-β1 in adipose tissues from lean mice *ex vivo* and in cultured adipocytes *in vitro* (Samad et al., 1997). In primary mouse lung fibroblasts and the Swiss 3T3 fibroblast cell line, TNF-α treatment also induces TGF-β1 expression at the transcriptional level through the activation of ERK (Sullivan et al., 2005), and AP-1 pathways (Sullivan et al., 2009). Inhibition of ERK with pharmaceutical inhibitors (PD98059 and U0126) blocked TNF-α-induced stabilization of TGF-β1 mRNA; overexpression of active MEK1, an upstream activator of ERK, instead enhances TGF-β1 mRNA stability (Sullivan et al., 2005). Furthermore, TNF-α was also found to increase nuclear levels of c-Jun and its binding to the DNA promoter region of TGF-β1. Accordingly, inhibition of AP-1 signaling attenuates upregulation of TGF-β1 induced by TNF-α in the Swiss 3T3 fibroblasts (Sullivan et al., 2009).

It remains relevant that if the oxidized low-density lipoprotein (ox-LDL), frequently found to increase in obesity, also induces the expression of TGF-β1. A cross-sectional study suggests that ox-LDL is significantly correlated with TGF-β1 in the sera of type 2 diabetic patients (Nakhjavani et al., 2009). Previous studies have demonstrated a causal role of ox-LDL in inducing TGF-β1 in human glomerular epithelial and mesangial cells (Ding et al., 1997; Song et al., 2005; Song et al., 2008) and porcine endothelial cells with potential implication in renal pathogenesis induced by TGF-β1 (Chatauret et al., 2014). With regards to the lung, ox-LDL has been shown to induce TGF-β1 expression in human alveolar epithelial cells that requires the activation of the Ras/ERK/PLTP pathway (Guo et al., 2012). In macrophages, ox-LDL was reported to mildly induce TGF-β1 production, which can be exaggerated by co-exposure with silica (Hou et al., 2019). Taken together, multiple mechanisms are likely to be involved in the induction of TGF-β1 by ox-LDL in the lung and cell-type specific effects are anticipated. However, it needs to be noted that many other studies fail to show the induction of TGF-β1 in the lung or peripheral circulation. The reasons could be due to differences in diet fat content, duration of HFD exposure, as well as the method of detection.

#### 3.2.2 Role of Transforming Growth Factor-β in the Development of Obesity

Increasing evidence support TGF-β as an important mediator for high-fat diet induced obesity and insulin resistance. Blockade of the TGF-β signaling through Smad3 knockout protects mice from high-fat diet induced obesity and insulin resistance (Tan et al., 2011; Tsurutani et al., 2011; Yadav et al., 2011). Conversely, overexpression of *glass bottom boat* (*gbb*), a drosophila homologue of mammalian TGF-β1, induces obesity and insulin resistance similarly as that induced by high-fat diet whereas inhibiting *gbb* leads to the opposite effects (Hong et al., 2016). These several lines of evidence point to a crucial role of TGF-β in regulating adipose tissue differentiation (adipogenesis) and energy metabolism. Indeed, altered expression of genes responsible for adipogenesis, fat accumulation, and fatty acid oxidation were observed in Smad3 deficient mice. A more detailed discussion of the crucial role of TGF-β/Smad signaling in obesity can be referred to a previous review (Tan et al., 2012).

#### 3.2.3 Role of Transforming Growth Factor-β in Obesity Related Pulmonary Fibrosis

The critical role of TGF-β in high-fat diet associated lung fibrosis has been assessed with the use of genetic modified animal models and pharmaceutical approaches targeting TGF-β/Smad3 signaling. Inhibition of TGF-β signaling through knockout of Smad3, the key mediator of TGF-β canonical pathway, successfully abolishes pulmonary fibrosis induced by high-fat diet (Tan et al., 2011; Yadav et al., 2011). In accord, the administration of TGF-β1 neutralizing antibody demonstrates similar protecting effects against HFD-induced peribronchial and perivascular fibrosis (Park et al., 2019). In addition, airway hyperreactivity and fibrosis associated with HFD are suggested to be attributed to enhanced airway TGF-β1 expression. One study showed that asthmatic patients exhibited elevated TGF-β1 in airways which correlated with the severity of asthmatics as well as infiltration of eosinophils and that the subepithelial airways fibrosis also correlated with the severity of asthmatics (Minshall et al., 1997), suggesting an important role of eosinophil-derived TGF-β in airway fibrosis, and AHR. Similar correlation between TGF-β and subepithelial fibrosis in asthmatic patients was reported and further, both eosinophils and fibroblasts were found to account for the increased TGF-β1 synthesis (Vignola et al., 1997). Other cell types including epithelial cells, macrophages, and neutrophils may also contribute to airway remodeling in asthmatics (Al-Alawi et al., 2014). In contrast, Jung et al. reported that mice with mild obesity induced by high-fat diet did not develop AHR or eosinophilic infiltration in the lung (Jung et al., 2013), suggesting that the severity of obesity may play a role in the development of AHR or fibrosis.

### 3.3 Monocyte Chemoattractant Protein-1

MCP-1 is a potent chemokine that induces the infiltration of macrophages into the site of inflammation. In obesity, adipose tissue produces increased levels of MCP-1 that is released into the peripheral blood. Elevated MCP-1 levels have been reported in both obese adults (Catalan et al., 2007) and obese children (Breslin et al., 2012). Further, obese children with lung fibrosis were reported to have significantly higher MCP-1 levels in the bronchoalveolar lavage (BAL) (Hartl et al., 2005). In an experimental fibrosis model, MCP-1 level was significantly increased between 3 and 10 days after bleomycin treatment. Targeting MCP-1 using overexpression of a mutant form of MCP-1, accordingly, attenuates pulmonary fibrosis induced by bleomycin in C57BL/6 mice (Inoshima et al., 2004). Despite these findings, evidence supporting the role of MCP-1 in lung fibrosis especially in the context of obesity remains scarce. More research is needed before any conclusion can be made.

### 3.4 Interleukins

Interleukins are a type of cytokines crucial for modulating inflammatory response and immune functions that are derived from various cell types including macrophages, lymphocytes, mast cells, fibroblasts, and epithelial cells, etc. (Akdis et al., 2016). Aberrant levels of cytokines from IPF patients has been reported and the role of interleukins in the pathogenesis of pulmonary fibrosis has been reviewed recently (She et al., 2021), among the upregulated interleukins in serum or bronchoalveolar lavage fluid (BALF) include IL-1β, IL-2, IL-8, IL-10, IL-12, IL-17A, and IL-33. While a common role in regulation inflammation exists, they act differently in terms of collagen synthesis or fibrosis. For example, the favorable group for pulmonary fibrosis includes IL-1β, IL-4, IL-6, IL11, IL-13, IL-17A, IL-15, and IL-33; in contrast, the other group with anti-fibrotic function has IL-7, IL-10, IL-12, and IL-27 (Borthwick, 2016; Steen et al., 2020; She et al., 2021). In obesity, both adipocytes and infiltrated inflammatory cells release interleukins in the adipose tissue and the blood stream (Bastard et al., 2006; Um et al., 2011; Tateya et al., 2013). Therefore, there is a potential role of interleukins in the IPF development associated with obesity.

## 4 Interaction of High-Fat Diet With Bleomycin-Induced Pulmonary Fibrosis

Of note, high-fat diet contributes to increased severity of experimental pulmonary fibrosis induced by bleomycin. A recent study shows that administration of palmitic acid (PA), one kind of saturated fatty acid, significantly increased pulmonary fibrosis in mice challenged with bleomycin compared to bleomycin challenged mice fed a standard diet (Chu et al., 2019). The interaction is likely due to increased apoptosis and endoplasmic reticulum (ER) stress in the lung epithelial cells. Downregulation of CD36, a fatty acid transporter, attenuated the effects of PA on apoptosis, and ER stress induction in the lung epithelial cells. These data suggest a link between epithelial lipotoxicity on the development of pulmonary fibrosis. Hegab et al. also reported that HFD delayed the resolution of lung fibrosis and alveolar repair following bleomycin administration in mice (Hegab et al., 2021). The epithelial repair, e.g., infiltration of alveolar type-2 cells and bronchioalveolar stem cell into the fibrotic foci, was dampened by HFD. The enhanced fatty acid oxidation due to high-fat diet is proposed and inhibition of FAO abolished HFD-induced a delay in alveolar repair and fibrosis resolution *in vivo*. It is noteworthy that the same group also reported that HFD increases the activation and number of alveolar type 2 cells in the lungs (Hegab et al., 2018). Such increase is suggested to be an indirect effect that is possibly linked with chronic inflammation. However, it remains elusive about the mechanisms underlying the impaired infiltration of alveolar type-2 cells and bronchioalveolar stem cells into fibrotic areas. The exploring of other cell types including the lung fibroblasts, and macrophages, etc., in the contribution of HFD to bleomycin-induced pulmonary fibrosis might be worthwhile for future studies.

## 5 Implication of Obesity in the Outcomes of Idiopathic Pulmonary Fibrosis Patients

Accumulating evidence suggest that obesity is an independent predictor for the outcomes of IPF patients. A higher body mass index (BMI) in IPF patients has also been recently shown to improve the mortality, although the overall morbidity is more prevalent in obese IPF patients (Mujahid et al., 2020). Similar paradoxical findings of obesity in IPF patients have also been reported in another IPF cohort conducted in the Appalachian area. An increase in BMI was found to predict better prognosis in IPF patients (Sangani et al., 2021). Acute exacerbation of IPF (AE-IPF) represents the leading cause of mortality in IPF patients. Recently, researchers in Japan evaluated the relationship between BMI and in-hospital mortality in patients with AE-IPF using a large retrospective cohort consisting of 14,783 patients. In those AE-IPF patients, the underweight subgroup showed higher mortality rate than the obese subgroup (Awano et al., 2021). Consistently, those IPF patients who experienced body weight loss (≥5%) within the first year of diagnosis show a worst prognosis compared to those without body weight loss (Nakatsuka et al., 2018). In general, these studies demonstrate a protective effect of obesity on the disease burden of IPF. In contrast, obesity seems to be adversely associated with the mortality of IPF patients who undergo bilateral lung transplant. The obese IPF patients have been reported to have increased waitlist and 90-days post-transplant mortality, i.e., those IPF patients with a BMI >30 kg/m^2^ are at 1.71 more risk of mortality within 3 months after bilateral lung transplant compared to those with BMIs from 18.5 to 30 kg/m^2^ (Gries et al., 2015). Similarly, another study showed that IPF patients who are obese (BMI >30 kg/m^2^) were 1.71 times more likely to die within 1 year compared to nonobese IPF patients (Lederer et al., 2009). These observations resemble the paradoxical effects of obesity on acute lung inflammation and injury and the outcomes (Zhi et al., 2016). The systematic low-grade inflammation might prime the lung and attenuate the immune responses resulting from insults from bacterial, viral, or chemical origin.

## 6 Concluding Remarks and Perspectives

**FIGURE 1:**
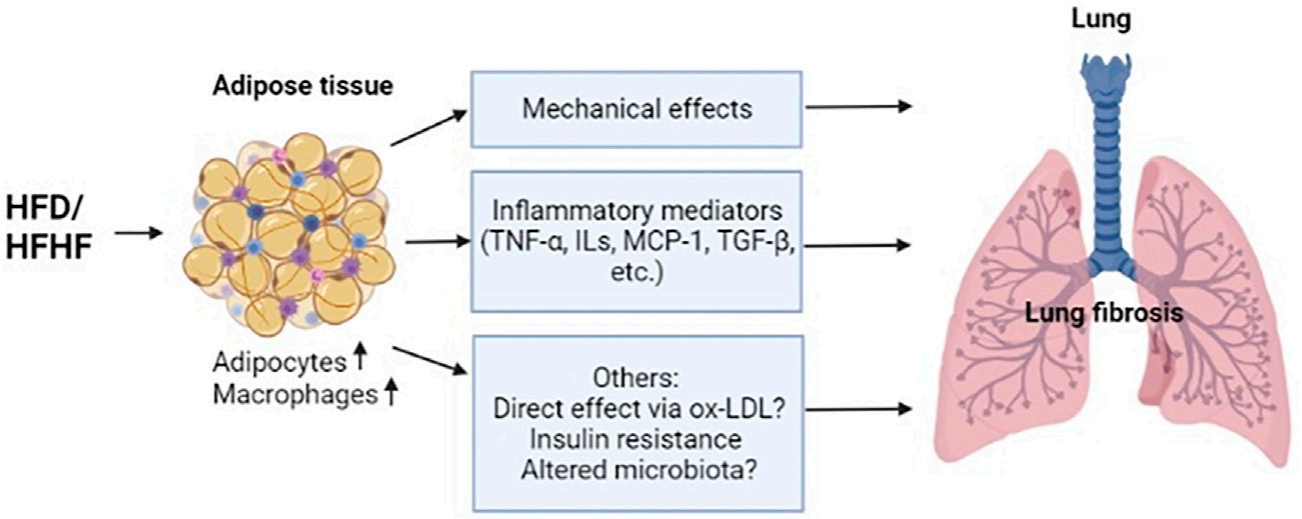
A Proposed model of obesity caused lung fibrosis.

Obesity is an increasing epidemic worldwide and it has considerable effects on the inflammatory infiltration and remodeling of the lung. A proposed model of the relationship between obesity and lung fibrosis is shown in Figure 1. High-fat diet induced inflammatory mediators (TNF-α, MCP-1, and TGF-β) from adipose tissues involving adipocytes and macrophages are important for the development of lung fibrosis. Mechanic effects of accumulating fat impairs lung function and induces fibrotic changes in the diaphragm (Buras et al., 2019), which may play a role in the fibrosis of the lung through inflammatory changes. Other factors related with obesity are likely being involved to promote lung fibrosis, such as a potential direct effect through oxidized LDL (Qian et al., 2021), indirect conditions such as insulin resistance (Park et al., 2019) and altered microbiota in the lung (Chioma et al., 2021). Inflamed lung also propagates the secretion of those mediators from diverse cell types locally, further leading to a transition towards profibrotic changes. Identification of inflammatory mediators that are important for both obesity and lung fibrosis might hold promise for the treatment or prevention of obesity-related lung fibrosis. Better understanding of the relationship between obesity and lung fibrosis may require further research using both a cell-specific approach and a systemic method to elucidate the roles of important inflammatory mediators/regulators implicated in both obesity and lung fibrosis, which may eventually lead to the identification of novel intervention or therapeutic strategies for lung fibrosis associated with obesity.

## Abbreviations

DIO, diet-induced obesity; HFD, high-fat diet; HFHF, high-fat high-fructose diet; IPF, idiopathic pulmonary fibrosis; MCP-1, monocyte chemoattractant protein-1; TGF-β, transforming growth factor-β; TNF-α, tumor necrosis factor-α.

## References

[B1] Akdis M. Aab A. Altunbulakli C. Azkur K. Costa R. A. Crameri R. (2016). Interleukins (From IL-1 to IL-38), Interferons, Transforming Growth Factor β, and TNF-α: Receptors, Functions, and Roles in Diseases. J. Allergy Clin. Immunol. 138, 984–1010. 10.1016/j.jaci.2016.06.033 27577879

[B2] Al-Alawi M. Hassan T. Chotirmall S. H. (2014). Transforming Growth Factor β and Severe Asthma: a Perfect Storm. Respir. Med. 108, 1409–1423. 10.1016/j.rmed.2014.08.008 25240764

[B3] Anand G. Katz P. O. (2010). Gastroesophageal Reflux Disease and Obesity. Gastroenterol. Clin. North. Am. 39, 39–46. 10.1016/j.gtc.2009.12.002 20202577

[B4] Awano N. Jo T. Yasunaga H. Inomata M. Kuse N. Tone M. (2021). Body Mass index and In-Hospital Mortality in Patients with Acute Exacerbation of Idiopathic Pulmonary Fibrosis. ERJ Open Res. 7, 00037–2021. 10.1183/23120541.00037-2021 34195254 PMC8236619

[B5] Baack M. L. Forred B. J. Larsen T. D. Jensen D. N. Wachal A. L. Khan M. A. (2016). Consequences of a Maternal High-Fat Diet and Late Gestation Diabetes on the Developing Rat Lung. PLoS One 11, e0160818. 10.1371/journal.pone.0160818 27518105 PMC4982689

[B6] Bastard J. P. Maachi M. Lagathu C. Kim M. J. Caron M. Vidal H. (2006). Recent Advances in the Relationship between Obesity, Inflammation, and Insulin Resistance. Eur. Cytokine Netw. 17, 4–12. 16613757

[B7] Borthwick L. A. (2016). The IL-1 Cytokine Family and its Role in Inflammation and Fibrosis in the Lung. Semin. Immunopathol. 38, 517–534. 10.1007/s00281-016-0559-z 27001429 PMC4896974

[B8] Breslin W. L. Johnston C. A. Strohacker K. Carpenter K. C. Davidson T. R. Moreno J. P. (2012). Obese Mexican American Children Have Elevated MCP-1, TNF-α, Monocyte Concentration, and Dyslipidemia. Pediatrics 129, e1180–6. 10.1542/peds.2011-2477 22473371

[B9] Bringardner B. D. Baran C. P. Eubank T. D. Marsh C. B. (2008). The Role of Inflammation in the Pathogenesis of Idiopathic Pulmonary Fibrosis. Antioxid. Redox Signal. 10, 287–301. 10.1089/ars.2007.1897 17961066 PMC2737712

[B10] Buechler C. Krautbauer S. Eisinger K. (2015). Adipose Tissue Fibrosis. World J. Diabetes 6, 548–553. 10.4239/wjd.v6.i4.548 25987952 PMC4434075

[B11] Buras E. D. Converso-Baran K. Davis C. S. Akama T. Hikage F. Michele D. E. (2019). Fibro-Adipogenic Remodeling of the Diaphragm in Obesity-Associated Respiratory Dysfunction. Diabetes 68, 45–56. 10.2337/db18-0209 30361289 PMC6302533

[B12] Catalán V. Gómez-Ambrosi J. Ramirez B. Rotellar F. Pastor C. Silva C. (2007). Proinflammatory Cytokines in Obesity: Impact of Type 2 Diabetes Mellitus and Gastric Bypass. Obes. Surg. 17, 1464–1474. 10.1007/s11695-008-9424-z 18219773

[B13] Cavalera M. Wang J. Frangogiannis N. G. (2014). Obesity, Metabolic Dysfunction, and Cardiac Fibrosis: Pathophysiological Pathways, Molecular Mechanisms, and Therapeutic Opportunities. Transl Res. 164, 323–335. 10.1016/j.trsl.2014.05.001 24880146 PMC4180761

[B14] Chatauret N. Favreau F. Giraud S. Thierry A. Rossard L. Le Pape S. (2014). Diet-induced Increase in Plasma Oxidized LDL Promotes Early Fibrosis in a Renal Porcine Auto-Transplantation Model. J. Transl Med. 12, 76. 10.1186/1479-5876-12-76 24655356 PMC3994364

[B15] Chiang D. J. Pritchard M. T. Nagy L. E. (2011). Obesity, Diabetes Mellitus, and Liver Fibrosis. Am. J. Physiol. Gastrointest. Liver Physiol. 300, G697–G702. 10.1152/ajpgi.00426.2010 21350183 PMC3094133

[B16] Chioma O. S. Hesse L. E. Chapman A. Drake W. P. (2021). Role of the Microbiome in Interstitial Lung Diseases. Front. Med. (Lausanne) 8, 595522. 10.3389/fmed.2021.595522 33604346 PMC7885795

[B17] Chow F. Y. Nikolic-Paterson D. J. Ozols E. Atkins R. C. Tesch G. H. (2005). Intercellular Adhesion Molecule-1 Deficiency Is Protective against Nephropathy in Type 2 Diabetic Db/db Mice. J. Am. Soc. Nephrol. 16, 1711–1722. 10.1681/ASN.2004070612 15857924

[B18] Chu S. G. Villalba J. A. Liang X. Xiong K. Tsoyi K. Ith B. (2019). Palmitic Acid-Rich High-Fat Diet Exacerbates Experimental Pulmonary Fibrosis by Modulating Endoplasmic Reticulum Stress. Am. J. Respir. Cel. Mol. Biol. 61, 737–746. 10.1165/rcmb.2018-0324OC PMC6890409 31461627

[B19] Deji N. Kume S. Araki S. Soumura M. Sugimoto T. Isshiki K. (2009). Structural and Functional Changes in the Kidneys of High-Fat Diet-Induced Obese Mice. Am. J. Physiol. Ren. Physiol. 296, F118–F126. 10.1152/ajprenal.00110.2008 18971213

[B20] Ding G. van Goor H. Ricardo S. D. Orlowski J. M. Diamond J. R. (1997). Oxidized LDL Stimulates the Expression of TGF-Beta and Fibronectin in Human Glomerular Epithelial Cells. Kidney Int. 51, 147–154. 10.1038/ki.1997.18 8995728

[B21] Distler J. H. Schett G. Gay S. Distler O. (2008). The Controversial Role of Tumor Necrosis Factor Alpha in Fibrotic Diseases. Arthritis Rheum. 58, 2228–2235. 10.1002/art.23645 18668576

[B22] Dixon A. E. Peters U. (2018). The Effect of Obesity on Lung Function. Expert Rev. Respir. Med. 12, 755–767. 10.1080/17476348.2018.1506331 30056777 PMC6311385

[B23] Fujita M. Shannon J. M. Morikawa O. Gauldie J. Hara N. Mason R. J. (2003). Overexpression of Tumor Necrosis Factor-Alpha Diminishes Pulmonary Fibrosis Induced by Bleomycin or Transforming Growth Factor-Beta. Am. J. Respir. Cel. Mol. Biol. 29, 669–676. 10.1165/rcmb.2002-0046OC 12816730

[B24] Ge X. N. Greenberg Y. Hosseinkhani M. R. Long E. K. Bahaie N. S. Rao A. (2013). High-fat Diet Promotes Lung Fibrosis and Attenuates Airway Eosinophilia after Exposure to Cockroach Allergen in Mice. Exp. Lung Res. 39, 365–378. 10.3109/01902148.2013.829537 24102347 PMC4474470

[B25] Gibbons M. A. MacKinnon A. C. Ramachandran P. Dhaliwal K. Duffin R. Phythian-Adams A. T. (2011). Ly6Chi Monocytes Direct Alternatively Activated Profibrotic Macrophage Regulation of Lung Fibrosis. Am. J. Respir. Crit. Care Med. 184, 569–581. 10.1164/rccm.201010-1719OC 21680953

[B26] Gries C. J. Bhadriraju S. Edelman J. D. Goss C. H. Raghu G. Mulligan M. S. (2015). Obese Patients with Idiopathic Pulmonary Fibrosis Have a Higher 90-day Mortality Risk with Bilateral Lung Transplantation. J. Heart Lung Transpl. 34, 241–246. 10.1016/j.healun.2014.09.031 25447567

[B27] Guo L. L. Chen Y. J. Wang T. An J. Wang C. N. Shen Y. C. (2012). Ox-LDL-induced TGF-β1 Production in Human Alveolar Epithelial Cells: Involvement of the Ras/ERK/PLTP Pathway. J. Cel. Physiol. 227, 3185–3191. 10.1002/jcp.24005 22034170

[B28] Hales C. M. Fryar C. D. Carroll M. D. Freedman D. S. Ogden C. L. (2018). Trends in Obesity and Severe Obesity Prevalence in US Youth and Adults by Sex and Age, 2007-2008 to 2015-2016. JAMA. 319, 1723–1725. 10.1001/jama.2018.3060 29570750 PMC5876828

[B29] Han H. Chung S. I. Park H. J. Oh E. Y. Kim S. R. Park K. H. (2021). Obesity-induced Vitamin D Deficiency Contributes to Lung Fibrosis and Airway Hyperresponsiveness. Am. J. Respir. Cel. Mol. Biol. 64, 357–367. 10.1165/rcmb.2020-0086OC 33296297

[B30] Hanson C. Rutten E. P. Wouters E. F. Rennard S. (2014). Influence of Diet and Obesity on COPD Development and Outcomes. Int. J. Chron. Obstruct Pulmon Dis. 9, 723–733. 10.2147/COPD.S50111 25125974 PMC4130708

[B31] Hartl D. Griese M. Nicolai T. Zissel G. Prell C. Reinhardt D. (2005). A Role for MCP-1/CCR2 in Interstitial Lung Disease in Children. Respir. Res. 6, 93. 10.1186/1465-9921-6-93 16095529 PMC1199626

[B32] Hegab A. E. Ozaki M. Meligy F. Y. Kagawa S. Ishii M. Betsuyaku T. (2018). High Fat Diet Activates Adult Mouse Lung Stem Cells and Accelerates Several Aging-Induced Effects. Stem Cel Res 33, 25–35. 10.1016/j.scr.2018.10.006 30308415

[B33] Hegab A. E. Ozaki M. Kagawa S. Fukunaga K. (2021). Effect of High Fat Diet on the Severity and Repair of Lung Fibrosis in Mice. Stem Cell Dev 30, 908–921. 10.1089/scd.2021.0050 34269615

[B34] Hong S. H. Kang M. Lee K. S. Yu K. (2016). High Fat Diet-Induced TGF-β/Gbb Signaling Provokes Insulin Resistance through the Tribbles Expression. Sci. Rep. 6, 30265. 10.1038/srep30265 27484164 PMC4971497

[B35] Hotamisligil G. S. Shargill N. S. Spiegelman B. M. (1993). Adipose Expression of Tumor Necrosis Factor-Alpha: Direct Role in Obesity-Linked Insulin Resistance. Science 259, 87–91. 10.1126/science.7678183 7678183

[B36] Hou X. Summer R. Chen Z. Tian Y. Ma J. Cui J. (2019). Lipid Uptake by Alveolar Macrophages Drives Fibrotic Responses to Silica Dust. Sci. Rep. 9, 399. 10.1038/s41598-018-36875-2 30674959 PMC6344530

[B37] Inoshima I. Kuwano K. Hamada N. Hagimoto N. Yoshimi M. Maeyama T. (2004). Anti-monocyte Chemoattractant Protein-1 Gene Therapy Attenuates Pulmonary Fibrosis in Mice. Am. J. Physiol. Lung Cel. Mol. Physiol. 286, L1038–L1044. 10.1152/ajplung.00167.2003 15064241

[B38] Jung S. H. Kwon J. M. Shim J. W. Kim D. S. Jung H. L. Park M. S. (2013). Effects of Diet-Induced Mild Obesity on Airway Hyperreactivity and Lung Inflammation in Mice. Yonsei Med. J. 54, 1430–1437. 10.3349/ymj.2013.54.6.1430 24142648 PMC3809850

[B39] Laurentius T. Raffetseder U. Fellner C. Kob R. Nourbakhsh M. Floege J. (2019). High-fat Diet-Induced Obesity Causes an Inflammatory Microenvironment in the Kidneys of Aging Long-Evans Rats. J. Inflamm. (Lond) 16, 14. 10.1186/s12950-019-0219-x 31289451 PMC6593534

[B40] Lederer D. J. Wilt J. S. D'Ovidio F. Bacchetta M. D. Shah L. Ravichandran S. (2009). Obesity and Underweight Are Associated with an Increased Risk of Death after Lung Transplantation. Am. J. Respir. Crit. Care Med. 180, 887–895. 10.1164/rccm.200903-0425OC 19608717 PMC2773915

[B41] Lee A. S. Mira-Avendano I. Ryu J. H. Daniels C. E. (2014). The burden of Idiopathic Pulmonary Fibrosis: an Unmet Public Health Need. Respir. Med. 108, 955–967. 10.1016/j.rmed.2014.03.015 24780718

[B42] Lee M. J. (2018). Transforming Growth Factor Beta Superfamily Regulation of Adipose Tissue Biology in Obesity. Biochim. Biophys. Acta Mol. Basis Dis. 1864, 1160–1171. 10.1016/j.bbadis.2018.01.025 29409985

[B43] Liu J. Y. Brass D. M. Hoyle G. W. Brody A. R. (1998). TNF-alpha Receptor Knockout Mice Are Protected from the Fibroproliferative Effects of Inhaled Asbestos Fibers. Am. J. Pathol. 153, 1839–1847. 10.1016/s0002-9440(10)65698-2 9846974 PMC1866331

[B44] Liu K. Zhou S. Liu J. Wang Y. Zhu F. Liu M. (2019). Silibinin Attenuates High-Fat Diet-Induced Renal Fibrosis of Diabetic Nephropathy. Drug Des. Devel. Ther. 13, 3117–3126. 10.2147/DDDT.S209981 PMC6718242 31695328

[B45] Marcelin G. Silveira A. L. M. Martins L. B. Ferreira A. V. Clément K. (2019). Deciphering the Cellular Interplays Underlying Obesity-Induced Adipose Tissue Fibrosis. J. Clin. Invest. 129, 4032–4040. 10.1172/JCI129192 31498150 PMC6763252

[B46] Minshall E. M. Leung D. Y. Martin R. J. Song Y. L. Cameron L. Ernst P. (1997). Eosinophil-Associated TGF-Beta1 mRNA Expression and Airways Fibrosis in Bronchial Asthma. Am. J. Respir. Cel. Mol. Biol. 17, 326–333. 10.1165/ajrcmb.17.3.2733 9308919

[B47] Miyake Y. Sasaki S. Yokoyama T. Chida K. Azuma A. Suda T. (2006). Dietary Fat and Meat Intake and Idiopathic Pulmonary Fibrosis: a Case-Control Study in Japan. Int. J. Tuberc. Lung Dis. 10, 333–339. 16562716

[B48] Miyazaki Y. Araki K. Vesin C. Garcia I. Kapanci Y. Whitsett J. A. (1995). Expression of a Tumor Necrosis Factor-Alpha Transgene in Murine Lung Causes Lymphocytic and Fibrosing Alveolitis. A Mouse Model of Progressive Pulmonary Fibrosis. J. Clin. Invest. 96, 250–259. 10.1172/JCI118029 7542280 PMC185196

[B49] Mujahid H. Sangani R. G. Catherman K. Patel Z. Zulfikar R. (2020). Obesity and Idiopathic Pulmonary Fibrosis (IPF): Defining Relationship, an Experience from a Rural Appalachian Tertiary Center. Am. J. Respir. Crit. Care Med. A3358. 10.1164/ajrccm-conference.2020.201.1_meetingabstracts.a3358

[B50] Nakatsuka Y. Handa T. Kokosi M. Tanizawa K. Puglisi S. Jacob J. (2018). The Clinical Significance of Body Weight Loss in Idiopathic Pulmonary Fibrosis Patients. Respiration 96, 338–347. 10.1159/000490355 30130749

[B51] Nakhjavani M. Esteghamati A. Asgarani F. Khalilzadeh O. Nikzamir A. Safari R. (2009). Association of Oxidized Low-Density Lipoprotein and Transforming Growth Factor-Beta in Type 2 Diabetic Patients: a Cross-Sectional Study. Transl. Res. 153, 86–90. 10.1016/j.trsl.2008.11.009 19138653

[B52] Naura A. S. Hans C. P. Zerfaoui M. Errami Y. Ju J. Kim H. (2009). High-fat Diet Induces Lung Remodeling in ApoE-Deficient Mice: an Association with an Increase in Circulatory and Lung Inflammatory Factors. Lab. Invest. 89, 1243–1251. 10.1038/labinvest.2009.98 19752857 PMC2784111

[B53] Oikonomou N. Harokopos V. Zalevsky J. Valavanis C. Kotanidou A. Szymkowski D. E. (2006). Soluble TNF Mediates the Transition from Pulmonary Inflammation to Fibrosis. PLoS One 1, e108. 10.1371/journal.pone.0000108 17205112 PMC1762410

[B54] Ortiz L. A. Lasky J. Hamilton R. F. Jr. Holian A. Hoyle G. W. Banks W. (1998). Expression of TNF and the Necessity of TNF Receptors in Bleomycin-Induced Lung Injury in Mice. Exp. Lung Res. 24, 721–743. 10.3109/01902149809099592 9839161

[B55] Ortiz L. A. Lasky J. Lungarella G. Cavarra E. Martorana P. Banks W. A. (1999). Upregulation of the P75 but Not the P55 TNF-Alpha Receptor mRNA after Silica and Bleomycin Exposure and protection from Lung Injury in Double Receptor Knockout Mice. Am. J. Respir. Cel. Mol. Biol. 20, 825–833. 10.1165/ajrcmb.20.4.3193 10101016

[B56] Park Y. H. Oh E. Y. Han H. Yang M. Park H. J. Park K. H. (2019). Insulin Resistance Mediates High-Fat Diet-Induced Pulmonary Fibrosis and Airway Hyperresponsiveness through the TGF-β1 Pathway. Exp. Mol. Med. 51, 1–12. 10.1038/s12276-019-0258-7 PMC6536500 31133649

[B57] Pessin J. E. Kwon H. (2012). How Does High-Fat Diet Induce Adipose Tissue Fibrosis? J. Investig. Med. 60, 1147–1150. 10.2310/JIM.0b013e318271fdb9 PMC3654516 23072903

[B58] Peters U. Dixon A. E. Forno E. (2018). Obesity and Asthma. J. Allergy Clin. Immunol. 141, 1169–1179. 10.1016/j.jaci.2018.02.004 29627041 PMC5973542

[B59] Piguet P. F. Vesin C. (1994). Treatment by Human Recombinant Soluble TNF Receptor of Pulmonary Fibrosis Induced by Bleomycin or Silica in Mice. Eur. Respir. J. 7, 515–518. 10.1183/09031936.94.07030515 7516893

[B60] Piguet P. F. Collart M. A. Grau G. E. Kapanci Y. Vassalli P. (1989). Tumor Necrosis Factor/cachectin Plays a Key Role in Bleomycin-Induced Pneumopathy and Fibrosis. J. Exp. Med. 170, 655–663. 10.1084/jem.170.3.655 2475571 PMC2189418

[B61] Piguet P. F. Collart M. A. Grau G. E. Sappino A. P. Vassalli P. (1990). Requirement of Tumour Necrosis Factor for Development of Silica-Induced Pulmonary Fibrosis. Nature 344, 245–247. 10.1038/344245a0 2156165

[B62] Qian G. Adeyanju O. Sunil C. Huang S. K. Chen S. Y. Tucker T. A. (2021). Dedicator of Cytokinesis 2 (DOCK2) Deficiency Attenuates Lung Injury Associated with Chronic High-Fat and High-Fructose Diet-Induced Obesity. Am. J. Pathol. S0002-9440 (21), 00474–0. 10.1016/j.ajpath.2021.10.011 PMC8883439 34767813

[B63] Redente E. F. Keith R. C. Janssen W. Henson P. M. Ortiz L. A. Downey G. P. (2014). Tumor Necrosis Factor-α Accelerates the Resolution of Established Pulmonary Fibrosis in Mice by Targeting Profibrotic Lung Macrophages. Am. J. Respir. Cel. Mol. Biol. 50, 825–837. 10.1165/rcmb.2013-0386OC PMC4068926 24325577

[B64] Salome C. M. King G. G. Berend N. (2010). Physiology of Obesity and Effects on Lung Function. J. Appl. Physiol. (1985) 108, 206–211. 10.1152/japplphysiol.00694.2009 19875713

[B65] Samad F. Yamamoto K. Pandey M. Loskutoff D. J. (1997). Elevated Expression of Transforming Growth Factor-Beta in Adipose Tissue from Obese Mice. Mol. Med. 3, 37–48. 10.1007/bf03401666 9132278 PMC2230108

[B66] Sangani R. G. Ghio A. J. Mujahid H. Patel Z. Catherman K. Wen S. (2021). Outcomes of Idiopathic Pulmonary Fibrosis Improve with Obesity: A Rural Appalachian Experience. South. Med. J. 114, 424–431. 10.14423/SMJ.0000000000001275 34215896 PMC9520755

[B67] Sgalla G. Iovene B. Calvello M. Ori M. Varone F. Richeldi L. (2018). Idiopathic Pulmonary Fibrosis: Pathogenesis and Management. Respir. Res. 19, 32. 10.1186/s12931-018-0730-2 29471816 PMC5824456

[B68] She Y. X. Yu Q. Y. Tang X. X. (2021). Role of Interleukins in the Pathogenesis of Pulmonary Fibrosis. Cell Death Discov 7, 52. 10.1038/s41420-021-00437-9 33723241 PMC7960958

[B69] Sime P. J. Marr R. A. Gauldie D. Xing Z. Hewlett B. R. Graham F. L. (1998). Transfer of Tumor Necrosis Factor-Alpha to Rat Lung Induces Severe Pulmonary Inflammation and Patchy Interstitial Fibrogenesis with Induction of Transforming Growth Factor-Beta1 and Myofibroblasts. Am. J. Pathol. 153, 825–832. 10.1016/s0002-9440(10)65624-6 9736031 PMC1853002

[B70] Song C. Y. Kim B. C. Hong H. K. Lee H. S. (2005). Oxidized LDL Activates PAI-1 Transcription through Autocrine Activation of TGF-Beta Signaling in Mesangial Cells. Kidney Int. 67, 1743–1752. 10.1111/j.1523-1755.2005.00271.x 15840021

[B71] Song C. Y. Kim B. C. Lee H. S. (2008). Lovastatin Inhibits Oxidized Low-Density Lipoprotein-Induced Plasminogen Activator Inhibitor and Transforming Growth Factor-Beta1 Expression via a Decrease in Ras/extracellular Signal-Regulated Kinase Activity in Mesangial Cells. Transl Res. 151, 27–35. 10.1016/j.trsl.2007.09.008 18061125

[B72] Song Y. Yu Y. Wang D. Chai S. Liu D. Xiao X. (2015). Maternal High-Fat Diet Feeding during Pregnancy and Lactation Augments Lung Inflammation and Remodeling in the Offspring. Respir. Physiol. Neurobiol. 207, 1–6. 10.1016/j.resp.2014.12.003 25500158

[B73] Sousa-Pinto B. Gonçalves L. Rodrigues A. R. Tomada I. Almeida H. Neves D. (2016). Characterization of TGF-β Expression and Signaling Profile in the Adipose Tissue of Rats Fed with High-Fat and Energy-Restricted Diets. J. Nutr. Biochem. 38, 107–115. 10.1016/j.jnutbio.2016.07.017 27736730

[B74] Spencer M. Yao-Borengasser A. Unal R. Rasouli N. Gurley C. M. Zhu B. (2010). Adipose Tissue Macrophages in Insulin-Resistant Subjects Are Associated with Collagen VI and Fibrosis and Demonstrate Alternative Activation. Am. J. Physiol. Endocrinol. Metab. 299, E1016–E1027. 10.1152/ajpendo.00329.2010 20841504 PMC3006260

[B75] Steen E. H. Wang X. Balaji S. Butte M. J. Bollyky P. L. Keswani S. G. (2020). The Role of the Anti-Inflammatory Cytokine Interleukin-10 in Tissue Fibrosis. Adv. Wound Care (New Rochelle) 9, 184–198. 10.1089/wound.2019.1032 32117582 PMC7047112

[B76] Sullivan D. E. Ferris M. Pociask D. Brody A. R. (2005). Tumor Necrosis Factor-Alpha Induces Transforming Growth Factor-Beta1 Expression in Lung Fibroblasts through the Extracellular Signal-Regulated Kinase Pathway. Am. J. Respir. Cel. Mol. Biol. 32, 342–349. 10.1165/rcmb.2004-0288OC 15653932

[B77] Sullivan D. E. Ferris M. Nguyen H. Abboud E. Brody A. R. (2009). TNF-Alpha Induces TGF-Beta1 Expression in Lung Fibroblasts at the Transcriptional Level via AP-1 Activation. J. Cel. Mol. Med. 13, 1866–1876. 10.1111/j.1582-4934.2009.00647.x PMC2855747 20141610

[B78] Tan C. K. Leuenberger N. Tan M. J. Yan Y. W. Chen Y. Kambadur R. (2011). Smad3 Deficiency in Mice Protects against Insulin Resistance and Obesity Induced by a High-Fat Diet. Diabetes 60, 464–476. 10.2337/db10-0801 21270259 PMC3028346

[B79] Tan C. K. Chong H. C. Tan E. H. Tan N. S. (2012). Getting 'Smad' about Obesity and Diabetes. Nutr. Diabetes 2, e29. 10.1038/nutd.2012.1 23449528 PMC3341711

[B80] Tateya S. Kim F. Tamori Y. (2013). Recent Advances in Obesity-Induced Inflammation and Insulin Resistance. Front. Endocrinol. (Lausanne) 4, 93. 10.3389/fendo.2013.00093 23964268 PMC3737462

[B81] Tsurutani Y. Fujimoto M. Takemoto M. Irisuna H. Koshizaka M. Onishi S. (2011). The Roles of Transforming Growth Factor-β and Smad3 Signaling in Adipocyte Differentiation and Obesity. Biochem. Biophys. Res. Commun. 407, 68–73. 10.1016/j.bbrc.2011.02.106 21356196

[B82] Tzanavari T. Giannogonas P. Karalis K. P. (2010). TNF-Alpha and Obesity. Curr. Dir. Autoimmun. 11, 145–156. 10.1159/000289203 20173393

[B83] Um J. Y. Rim H. K. Kim S. J. Kim H. L. Hong S. H. (2011). Functional Polymorphism of IL-1 Alpha and its Potential Role in Obesity in Humans and Mice. PLoS One 6, e29524. 10.1371/journal.pone.0029524 22216303 PMC3246492

[B84] Vedova M. C. D. Soler Garcia F. M. Muñoz M. D. Fornes M. W. Gomez Mejiba S. E. Gómez N. N. (2019). Diet-Induced Pulmonary Inflammation and Incipient Fibrosis in Mice: a Possible Role of Neutrophilic Inflammation. Inflammation 42, 1886–1900. 10.1007/s10753-019-01051-9 31359324

[B85] Vignola A. M. Chanez P. Chiappara G. Merendino A. Pace E. Rizzo A. (1997). Transforming Growth Factor-Beta Expression in Mucosal Biopsies in Asthma and Chronic Bronchitis. Am. J. Respir. Crit. Care Med. 156, 591–599. 10.1164/ajrccm.156.2.9609066 9279245

[B86] Yadav H. Quijano C. Kamaraju A. K. Gavrilova O. Malek R. Chen W. (2011). Protection from Obesity and Diabetes by Blockade of TGF-β/Smad3 Signaling. Cel. Metab 14, 67–79. 10.1016/j.cmet.2011.04.013 PMC3169298 21723505

[B87] Yu H. Z. Wu Q. Du Z. Z. Sun X. Wang C. Z. Li L. (2013). High-Fat Diet Induces Pulmonary Fibrosis in Rats and Inhibitory Effects of N-Acetylcysteine. Zhonghua Yi Xue Za Zhi 93, 3547–3550. 24521900

[B88] Zaman T. Lee J. S. (2018). Risk Factors for the Development of Idiopathic Pulmonary Fibrosis: A Review. Curr. Pulmonol Rep. 7, 118–125. 10.1007/s13665-018-0210-7 31588408 PMC6777743

[B89] Zhi G. Xin W. Ying W. Guohong X. Shuying L. (2016). "Obesity Paradox" in Acute Respiratory Distress Syndrome: Asystematic Review and Meta-Analysis. PLoS One 11, e0163677. 10.1371/journal.pone.0163677 27684705 PMC5042414

